# Resting-State Beta-Band Recovery Network Related to Cognitive Improvement After Stroke

**DOI:** 10.3389/fneur.2022.838170

**Published:** 2022-02-25

**Authors:** Sandra Pusil, Lucía Torres-Simon, Brenda Chino, María Eugenia López, Leonides Canuet, Álvaro Bilbao, Fernando Maestú, Nuria Paúl

**Affiliations:** ^1^Department of Experimental Psychology, Universidad Complutense de Madrid, Madrid, Spain; ^2^Institute of Neuroscience, Autonomous University of Barcelona, Barcelona, Spain; ^3^National Centre for Brain Injury Treatment, Centro de Referencia Estatal de Atención Al Daño Cerebral (CEADAC), Madrid, Spain

**Keywords:** stroke, functional connectivity (FC), MEG (magnetoencephalography), cognitive performance, neuropsychological rehabilitation

## Abstract

**Background:**

Stroke is the second leading cause of death worldwide and it causes important long-term cognitive and physical deficits that hamper patients' daily activity. Neuropsychological rehabilitation (NR) has increasingly become more important to recover from cognitive disability and to improve the functionality and quality of life of these patients. Since in most stroke cases, restoration of functional connectivity (FC) precedes or accompanies cognitive and behavioral recovery, understanding the electrophysiological signatures underlying stroke recovery mechanisms is a crucial scientific and clinical goal.

**Methods:**

For this purpose, a longitudinal study was carried out with a sample of 10 stroke patients, who underwent two neuropsychological assessments and two resting-state magnetoencephalographic (MEG) recordings, before and after undergoing a NR program. Moreover, to understand the degree of cognitive and neurophysiological impairment after stroke and the mechanisms of recovery after cognitive rehabilitation, stroke patients were compared to 10 healthy controls matched for age, sex, and educational level.

**Findings:**

After intra and inter group comparisons, we found the following results: (1) Within the stroke group who received cognitive rehabilitation, almost all cognitive domains improved relatively or totally; (2) They exhibit a pattern of widespread increased in FC within the beta band that was related to the recovery process (there were no significant differences between patients who underwent rehabilitation and controls); (3) These FC recovery changes were related with the enhanced of cognitive performance. Furthermore, we explored the capacity of the neuropsychological scores before rehabilitation, to predict the FC changes in the brain network. Significant correlations were found in global indexes from the WAIS-III: Performance IQ (PIQ) and Perceptual Organization index (POI) (i.e., Picture Completion, Matrix Reasoning, and Block Design).

## Introduction

Stroke is considered the second leading cause of death and the third leading cause of disability worldwide (1). It is a heterogeneous pathology with diverse clinical manifestations due to its possible etiologies (i.e., hemorrhagic, or ischemic), locations (i.e., different vascular vessels or arteries), and size of the lesion (2, 3). However, most stroke survivors suffer from different degrees of cognitive disabilities (4–6). These patients may have damage in general cognitive performance with important functional disability, which has been broadly reported in the scientific literature (7–9). Although stroke tends to impact on attention and executive function compared with its impact on memory, a malfunction in these cognitive domains could worsen the performance in other cognitive areas (3, 4, 6, 10). In any case, it is important to highlight the role of non-pharmacological rehabilitation especially neuropsychological rehabilitation (NR) in order to improve cognitive abilities and daily functions (10, 11). Neuropsychological rehabilitation is a systematic therapeutic activity oriented functionally based on the assessment and understanding of the cognitive deficits, emotional disturbances, disruptive behaviors, and functional disorders of patients (12, 13), and includes interventions that might be compensatory, educational, or restorative (10).

According to some authors and approaches post-stroke deficits have long been considered to be fundamentally associated with the location of the lesion (14). This could be particularly true for sensorimotor or language deficits, which are closely related to the damage to the specific eloquent cortex. However, it has been shown that although structural damage from stroke is usually focal, remote disturbances may occur in brain distant regions from the primary area of damage (15, 16). This phenomenon was previously associated with the concept of diaschisis, but it is currently explained by the disruption of structural and functional connectivity (FC) between brain areas (17). This way of understanding the functioning of the brain gives a crucial role to NR in the process of cognitive recovery, since it allows a holistic management of cognitive impairment in contrast to other more goal-oriented therapies.

In this context, as the restoration of FC precedes or accompanies in most cases, cognitive and behavioral recovery in stroke patients (18, 19), understanding the electrophysiological signatures underlying stroke recovery mechanisms is a crucial scientific goal. This information could help the clinical community to anticipate and modify NR programs to achieve a more effective cognitive recovery, and consequently, improve patients' quality of life. With this purpose, in the present study we used the magnetoencephalography (MEG), a neurophysiological technique that allows a comprehensive analysis of brain dynamics (20, 21). While the functional magnetic resonance image (fMRI) is intrinsically limited by the hemodynamic response, MEG directly measures cortical neural activity. That means that the modified vasomotor reactivity and neurovascular uncoupling in stroke easily affects the blood oxygen level-dependent (BOLD) response but leaves the MEG signal intact (22). The study of MEG signatures is well-established for early detection and prognosis in neurodegenerative disorders, such as multiple sclerosis (23) or Alzheimer's disease (24). Moreover, MEG has previously been used to demonstrate the disruption and recovery of functional networks, and even its relationship with cognitive improvement after undergoing a NR program in acquired brain pathologies such as stroke (22, 25, 26) or traumatic brain injury (TBI) (13, 27). Thanks to the relevance of the neurophysiological changes found in previous literature, it seems plausible that MEG may provide interesting information about how NR may induce specific cognitive recovery in stroke patients.

According to the aforementioned antecedents, the aims of the present exploratory study are: (1) to understand the cognitive improvement achieved in stroke patients that received NR; (2) to explore the possible neurophysiological mechanisms underlying the recovery process, by using FC on frequency bands obtained with MEG; and (3) to evaluate if these neurophysiological changes are related with cognitive improvement. For this purpose, we carried out a longitudinal study in which a sample of 10 stroke patients (stroke patients) were examined at two different time points. The first was before NR (from now on we will say *pre-condition*), and the second was after NR (from now on we will say *post-condition*). At both time points patients were cognitively evaluated and underwent resting-state MEG recordings. Moreover, to understand the degree of cognitive and neurophysiological disruption after stroke, and the recovery mechanisms in stroke patients who were enrolled on the NR, data for a control group were included with 10 healthy controls paired in age, sex, and educational level.

## Materials and Methods

### Participants

The total dataset consisted of 20 subjects: 10 stroke patients (2 females/8 males; mean age 44.9 ± 8.94; mean level of education 4.44 ± 0.97) and 10 healthy controls (2 females/8 males; mean age 43 ± 12.72; mean level of education 4.78 ± 0.67). The mean time from the onset of the stroke to the start of the study was 6.3 months, and the rehabilitation program lasted 7 months. The patient's lesions were both ischemic (i.e., infarction; *n* = 5) and hemorrhagic (i.e., intracerebral hemorrhage; *n* = 5) and the stroke was located in different brain areas (for patient detailed descriptive data see Table 1). To be enrolled in the study, patients had to be diagnosed with a first-ever stroke, showing a compatible lesion observed on computerized tomography (CT) or magnetic resonance imaging (MRI). Although initially, after the stroke some patients showed loss of consciousness [as reported in Table 1 with the Glasgow Coma Scale (28)], at the beginning of the study all patients were neurologically stable without alterations in consciousness or alertness, and none of them showed epileptiform discharges on MEG recordings.

**Table 1:** Clinical and sociodemographic characteristics of the patients.

**Patient**	**Age**	**Sex**	**Education**	**GCS**	**Stroke etiology**	**Stroke lesion**
1	44	M	3	12	Ischemia	Right fronto-parietal
2	45	F	5	7	Ischemia	Right middle cerebral artery
3	47	M	5	9	Ischemia	Left middle cerebral artery
4	47	M	4	12	Ischemia	Right middle and anterior cerebral arteries
5	60	M	3	12	Ischemia	Left middle cerebral artery
6	28	F	4	7	Hemorrhage	Thalamus and left basal ganglia
7	35	M	4	8	Hemorrhage	Right intraparenchymal
8	41	M	5	7	Hemorrhage	Left basal ganglia
9	49	M	6	9	Hemorrhage	Right basal ganglia
10	53	M	5	9	Hemorrhage	Left thalamus
*N* = 10	44.9 ± 8.9	8 M/2 F	4.4 ± 0.9	9.2 ± 2.1	5 isch/5 hem	

*Education (1, illiterate/functional illiterate; 2, elemental studies; 3, school graduate; 4, high school studies; 5, university graduate studies; 6, university post-graduate studies)*.

Exclusion criteria were the following: a stroke involving the brainstem or cerebellum, a diagnosis of neurological or psychiatric diseases other than stroke, and a history of TBI, drug, or alcohol abuse.

Patients were recruited from the National Brain Injury Rehabilitation Center and from Lescer Brain Injury Rehabilitation Center (Madrid, Spain), and all of them were enrolled in a NR program. Healthy controls were matched with patients for age, sex, and education level, and they did not have a previous history of psychiatric or neurological disorders.

As previously mentioned, patients underwent MEG recordings and neuropsychological evaluation in two different moments: (1) Pre-condition (Pre): at the beginning of the study, before NR program; and (2) Post-condition (Post): after completing the NR program. In the case of healthy controls, both data, neuropsychological and neurophysiological, were obtained only once, at the beginning of the study.

### Ethics Statement

Methods were carried out in accordance with approved guidelines and regulations. The study was approved by the National Brain Injury Rehabilitation Center Ethics Committee (Madrid), and all participants or legal representatives signed a written informed consent prior to participation.

### Neuropsychological Assessment

All participants underwent a comprehensive neuropsychological evaluation with the aim to identify their cognitive status in multiple cognitive domains (attention, memory, language, executive functions, and visuospatial abilities) as well as their functional performance. The extensive neuropsychological assessment included: the Wechsler Adult Intelligence Scale III (WAIS III) (29), the Brief Test of Attention (BTA) (30), the Trail Making Test (TMT) (31), the Stroop Color Word Test (32, 33), the Wisconsin Card Sorting Test (WCST) (34), the Tower of Hanoi (35), the Zoo Map Test [from the Behavioral Assessment of the Dysexecutive Syndrome (36)], the Boston Naming Test (BNT) (37), the Digit Span Test [Wechsler Memory Scale III (29)], the Visual Span Test [WMS-III; (29)], Logical Memory and Visual Reproduction [WMS-III (29)], the Phonemic and Semantic Fluency [Controlled Oral Word Association Test, COWAT (38)], the Five Digit Test [FDT (39)], the Dysexecutive Questionnaire [DEX (36)], and the Patient Competency Rating Scale [PCRS (40)].

### Neuropsychological Rehabilitation Program

All stroke patients received an integrated treatment based on the holistic-comprehensive model proposed by Ben-Yishay and Diller (41). This program consists of 1 h/day of occupational therapy, 1 h/2 days/week of neuropsychological therapy, and 2 h/day of group cognitive therapy (memory and executive function/social skills). Neuropsychological therapy aimed to improve attention, working memory, learning, memory and problem solving/executive functions, and emotional-behavioral problems, through evidence-based techniques that included both restorative and compensatory strategies. Neuropsychological treatment goals in each case were defined to achieve maximum cognitive independence in daily living. In addition, patients underwent 1 h of physiotherapy and half an hour of speech therapy, in those cases that needed it. This rehabilitation plan met the following requirements: (1) agreed by the family and all professionals involved; (2) formulated in a specific and operational manner (3) focused on meaningful goals for the patients that allow them to achieve greater personal autonomy, community integration, and adaptation to their deficits; and (4) reviewed monthly. In addition, all patients attended psychotherapy sessions to help them in the process of accepting their new situation.

### Magnetoencephalographic Recordings

Magnetic fields were recorded using a 148-channel whole-head magnetometer (4D-MAGNES_2500 WH, 4-D Neuroimaging) confined in a magnetically shielded room at the Universidad Complutense of Madrid (Spain). Fields were measured during a 2-min resting-state eyes-closed condition and were sampled at a frequency rate of 618.17 Hz. Ocular, cardiac, muscular, and jump artifacts were identified first, by a visual inspection of an expert in MEG, and then removed using ICA (42) in Brainstorm software (43). Then, clean data were segmented into 4 s trial length, with a minimum of 20 artifact-free segments for each subject. The MEG data were filtered in the classical frequency bands: delta (2–4 Hz), theta (4–8 Hz), alpha (8–12 Hz), beta (12–30 Hz), and gamma (30–45 Hz) for further analysis.

### Source Reconstruction and Connectivity Analysis

To reconstruct MEG sources, we used the default anatomy (15,000 vertices) of the MNI/Colin27 brain (44) in Brainstorm. This template was warped according to the polhemus points (nasion and both preauricular) acquired during the head digitalization to obtain a better approximation of the real shape of the subject's head. The overlapping sphere model was calculated as the forward modeling of MEG measures. Next, a noise covariance matrix was calculated to estimate noise level in the MEG recordings. Sources were reconstructed using the weighted Minimum Norm Estimation (wMNE) (45). Weighted Minimum Norm Estimation is well-suited for the estimation of large-scale FC networks, since it addresses the problem of volume conduction, reducing the correlations of spurious signals (46, 47). Magnetoencephalography sources were grouped into 68 anatomical regions of interest (ROI) based on Brainstorm atlas Desikan-Killiany (48). For more details about the brain areas used, referred to Supplementary Material for Supplementary Table 1. We selected the mean as the representative time series for each brain area delimited with the aforementioned atlas.

**Figure 1:**
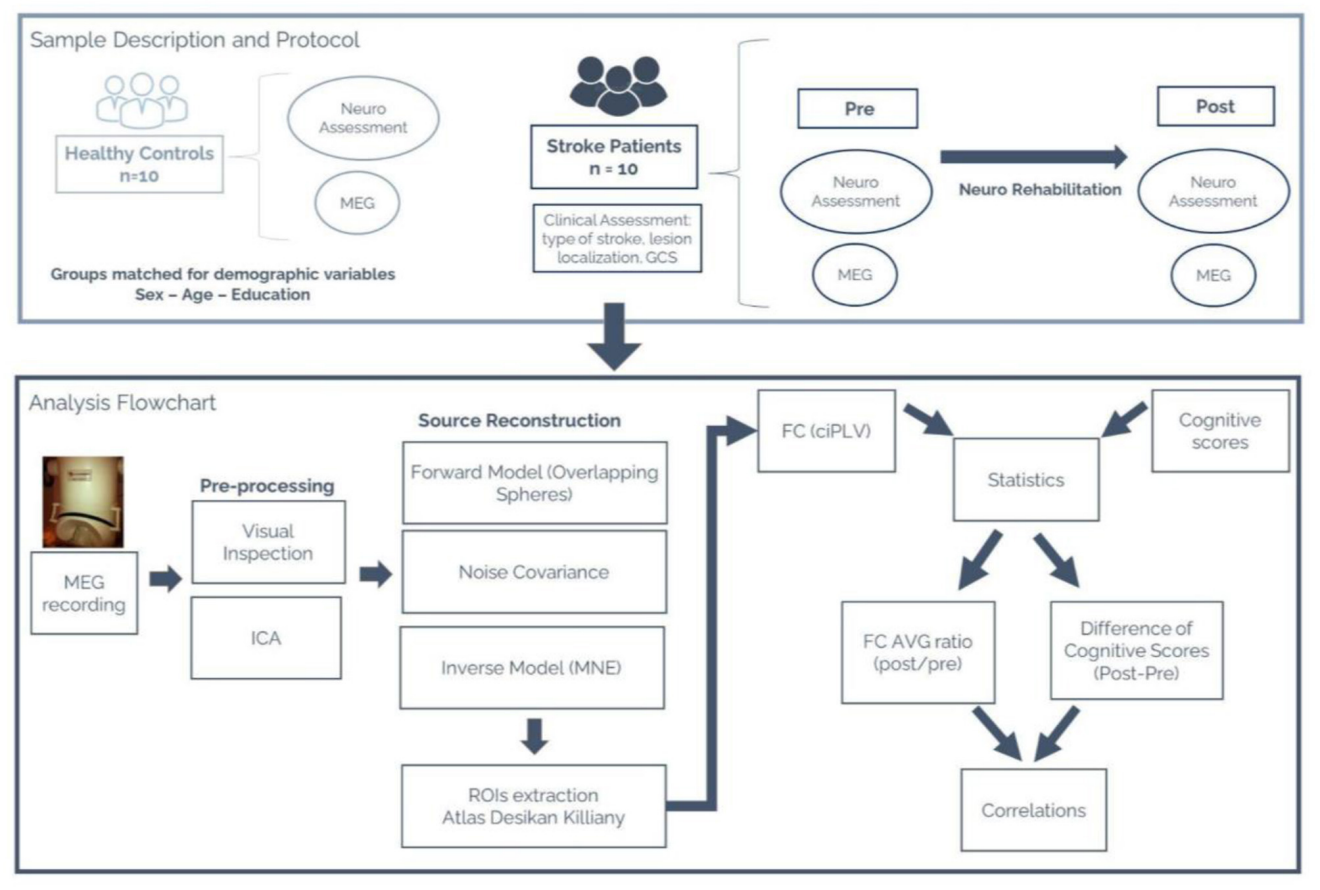
(Top) Sample description (stroke patients and healthy control groups). Description of the protocol followed by each group and the variables collected for this study. (Bottom) MEG Analysis flowchart. Sequential pipeline of the analysis performed on the MEG data.

Functional connectivity was assessed using the corrected version of the imaginary phase locking value (ciPLV), a phase synchronization measure that evaluates the distribution of phase differences extracted from each of two sensor time series (49, 50). Corrected version of the imaginary phase locking value (Equation 1) was proposed by Bruña et al. (50) to remove the contribution of the zero phase differences of PLV. Thus, this measure is insensitive to zero-lag effects, and it is corrected to remove the instantaneous phase contribution, which could be mainly due to volume conduction.


(1) $$\begin{array}{l} ciPLV_{X,Y}\left(t\right)=\;\frac{\frac{1\;}{T}I\left\{e^{-i\left(\phi_{X}\left(t\right)-\phi_{Y}\left(t\right)\right)}\right\}}{\sqrt{1-(\frac{1}{T}R\left\{e^{-i\left(\phi_{X}\left(t\right)-\phi_{Y}\left(t\right)\right)}\right\}{)}^{2}}} \end{array}$$

where φ_x_ and φ_y_ represent the phases of each of the two-time series and 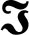 stands for the imaginary part of the numerator and 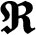 the real part in the denominator. See Figure 1 for the analysis flow chart of the MEG data.

### Statistical Analyses

This study aims to find the possible neurophysiological substrates of the recovery network underlying the cognitive enhancement found stroke patient's sample in the post condition (after NR), by using cognitive tests, functional scales, and FC measures. In this context, we performed exploratory analyses with the data obtained from stroke patients and healthy controls. The analysis of demographic data showed that there were no statistical differences in age, sex, and level of education between patients and controls (*p* > 0.05), so we did not include them as confounding variables for the following explorations. Non-parametric tests were used for all comparisons because variables were non-normally distributed and because of the small sample size. Specifically, the Mann-Whitney U test was used for between groups analyses (stroke patients vs. healthy controls) and Wilcoxon paired test for within-group comparison (Pre vs. Post conditions in the stroke patients' group). In the case of neuropsychological variables, significant results were considered with a *p*-value < 0.05 after applying false discovery rate (FDR) corrected for multiple comparisons. For FC data, a total of 10,000 permutations were used for each significant FC link, and results were considered significant with a *p*-value <0.005 after applying FDR (51). Finally, with the aim to explore the relationships between FC and cognition, Spearman's correlation analysis was employed. For all analyses the Matlab Statistical Toolbox was used.

## Results

### Cognitive Changes After Neuropsychological Rehabilitation

**Figure 2:**
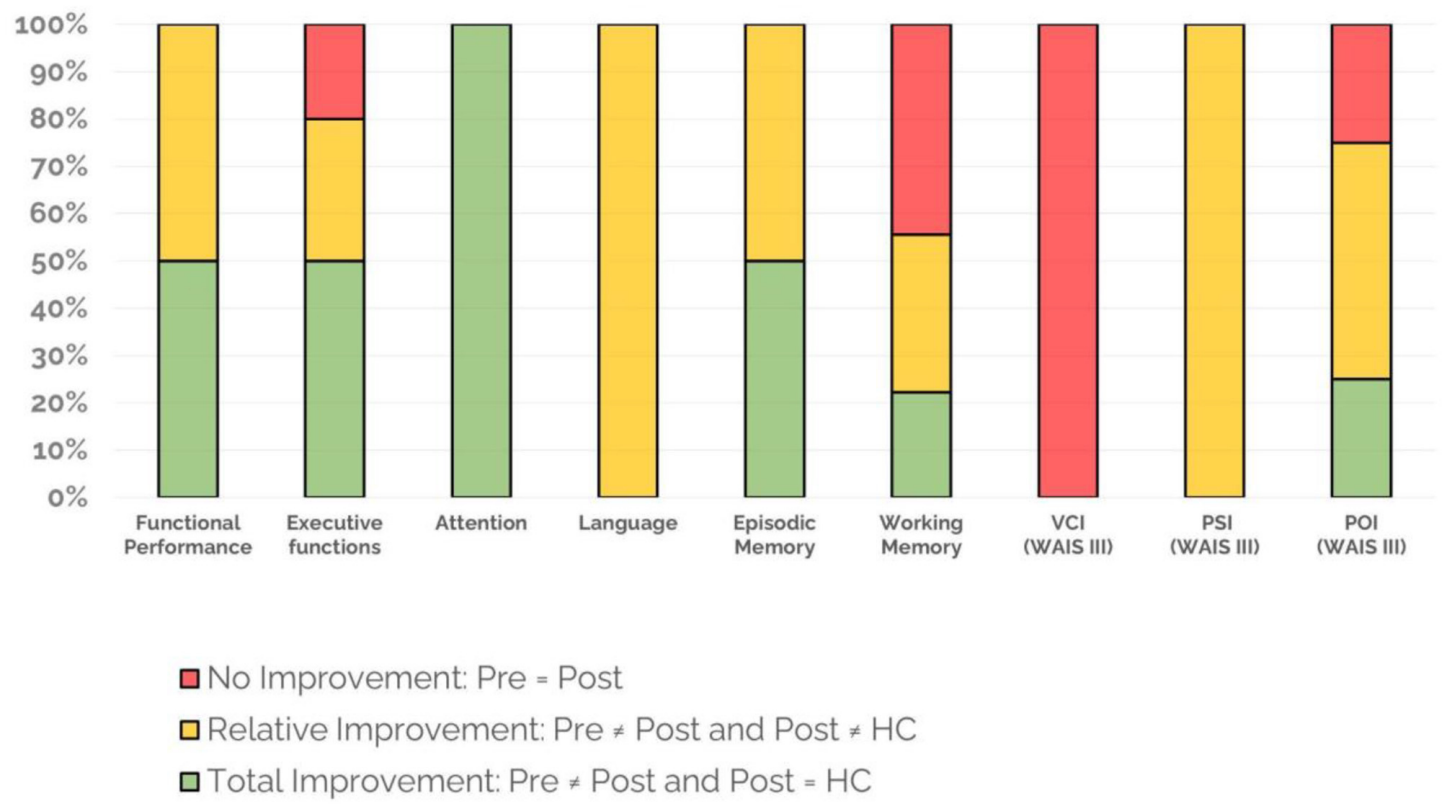
Neuropsychological tests' changes after NR. Functional Performance (4 scores: DEX and PCRS); Executive Functions (10 scores: WCST, Tower of Hanoi, FDT, and TMT); Attention (1 score: Brief Test of Attention); Language (2 scores: BNT and FAS); Episodic Memory (4 scores: WMS-III); Working Memory (9 scores: WAIS-III and WMS-III). The last three columns correspond to cognitive indices of WAIS-III, including all their subtests: VCI (verbal comprehension, 4 scores); PSI (speed processing, 3 scores); and POI (perceptual organization, 4 scores). Percentages were calculated intra-domain. Test scores included. DEX, DEX-family, DEX-difference; PCRS, PCRS-family, PCRS-difference; WCST, WCST-categories, WCST-conceptual, WCST-persevering; Tower of Hanoi, TH-3D-time, TH-3D-movements, TH-4D-time, TH-4D-movements; Five Digit Test, FDT-switching, FDT-flexibility; Brief Test of Attention, BTA-total score; BNT, BNT-total score; FAS, FAS-total score; WMS-III-episodic-memory, WMS-III-logical memory 1, WMS-III-logical memory 2, WMS-III-visual reproduction 1, WMS-III-visual reproduction 2; WMS-working memory, WMS-III-forward digit span, WMS-III-backward digit span, WMS-III-forward visual span, WMS-III-backward visual span; WAIS-working memory, WAIS-digit span, WAIS-arithmetic, WAIS-letter number sequencing, WAIS-working memory index; WAIS-VCI, WAIS-vocabulary, WAIS-information, WAIS-similarities, WAIS-verbal comprehension index; WAIS-PSI, WAIS-symbol search, WAIS-digit symbol, WAIS-processing speed index; WAIS-POI, WAIS-block design, WAIS-matrix reasoning, WAIS-picture completion; WAIS-perceptual organization index.

As described before, the patients underwent a comprehensive neuropsychological evaluation before and after the NR program. From the total of battery tests, those scores with at least nine reported patients (46 scores in total) were included for the statistical analysis. Pre-condition results indicated that stroke patients performed significantly worse compared with healthy controls in all cognitive domains (*p* < 0.05). Comparing pre and post conditions in the stroke patients' group, results showed an important cognitive improvement with 33 scores (72% of the 46 total scores) significantly different between conditions. Of these, 21 scores (46% of the 46 total scores), could be considered *relatively enhanced* since in the post-condition, they were significantly different to those corresponding to the healthy controls (see Figure 2). The remaining 12 scores (26% of the 46 total scores) from the post-condition did not show significant differences with the healthy control group, indicating a *total improvement*. To simplify the interpretation of these results, all scores were clustered into several aggregated groups depending on different cognitive domains: *Functional performance*, 4 scores (of DEX and PCRS); *Executive Functions*, 10 scores (of WCST, Tower of Hanoi, FDT and TMT); *Attention*, 1 score (of Brief Test of Attention); *Language*, 2 scores (of BNT and FAS); *Episodic Memory*, 4 scores (of WMS-III) and *Working Memory*, 9 scores (of WMS-III and WAIS-III). The last three columns of Figure 2 correspond to the cognitive index of WAIS-III, including all their subtests: Verbal Comprehension Index (VCI, 4 scores); Processing Speed Index (PSI, 3 scores), and Perceptual Organization Index (POI, 4 scores). In addition, the three general indices of WAIS-III were also included [Verbal IQ, Performance IQ (PIQ), and Full-Scale IQ], as well as subtests Picture Arrangement and Comprehension. In summary, we found that all cognitive domains of the stroke patients' group were fully or partially enhanced in the post-condition (see Figure 2). It is important to note that the VCI (that includes the WAIS-III index and all its subtests), was a cognitive domain without improvement in any of the four measures; and the four scores maintained significant differences when comparing the second scores of stroke patients with the healthy controls scores. However, it is important to know that this index, already in the pre-condition, shows a normal score (103.5) unlike the results of the other cognitive index of the WAIS-III (WMI 91.9; PSI 80.3; PRI 82.4) and the others all cognitive tests. In addition, their result in the post-condition was 105, although it continues to be statistically different from the healthy control group (117.6).

### Functional Connectivity Disruption After Stroke: Differences Between Stroke Patients and Healthy Controls

**Figure 3:**
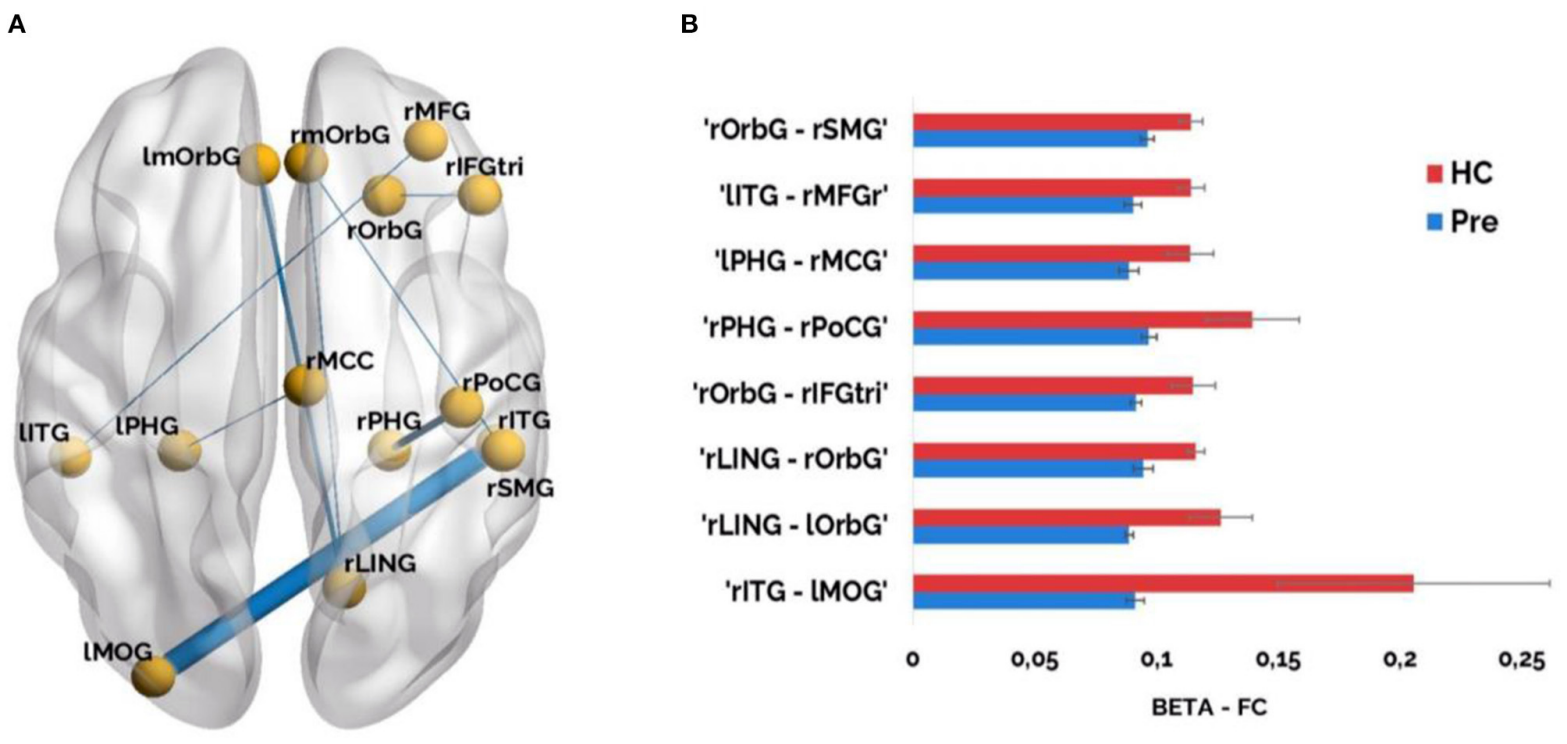
**(A)** Significant FC results (*p* < 0.005, corrected) in the beta band (12–30 Hz) when comparing healthy controls vs. stroke patients in the pre-condition. Line thickness of significant links is proportional to FC values (a higher value corresponds to thicker lines, and vice versa); **(B)** Significant FC links in the beta band are represented as bar graphs. Red color represents higher connectivity values for healthy controls compared to stroke patients and blue color illustrates lower connectivity values for stroke patients compared to healthy controls. ROIs included: rmOrbG, Right Medial Orbito Frontal Gyrus; lmOrbG, Left Medial Orbito Frontal Gyrus; rMFG, Right Middle Frontal Gyrus; rOrbG, Lateral Orbito Frontal Gyrus; rIFGtri, Right Inferior Frontal Parstriangularis; lITG, Left Inferior Temporal Gyrus; rITG, Right Inferior Temporal Gyrus; lPHG, Left Parahippocampal Gyrus; rPHG, Right Parahippocampal Gyrus; rMCC, Right Posterior Cingulate Gyrus; rPoCG, Right Postcentral Gyrus; rSMG, Right Supramarginal Gyrus; rLING, Right Lingual Cortex; lMOG, Left Lateral Occipital Gyrus.

In order to assess the possible disruption of the patients' network due to the stroke, their FC in the pre-condition was compared with the FC of the healthy controls. Stroke patients exhibited significant FC reduction in the beta band (*p* < 0.005, FDR corrected) that comprised intra and inter-hemispheric connections (Figure 3). No significant results were found in other frequency bands.

### Functional Connectivity After Rehabilitation: The Recovery Network

**Figure 4:**
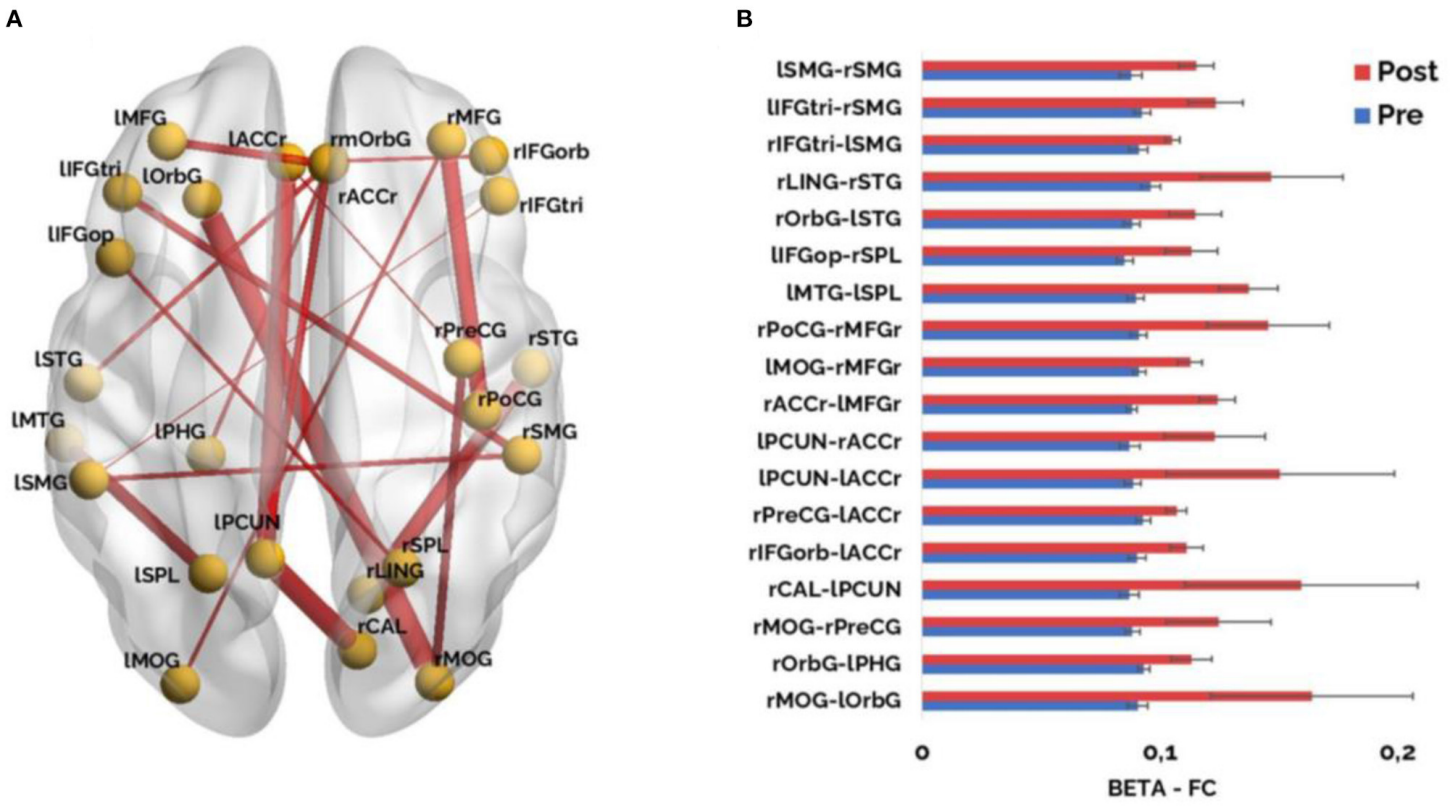
**(A)** Significant FC results (*p* < 0.005, corrected) in the beta band (12–30 Hz) when comparing within the stroke patients' group, the pre-condition and the post- condition. Line thickness of significant links is proportional to FC values (a higher value corresponds to thicker lines, and vice versa); **(B)** Significant functional connectivity links in the beta band are represented as bar graphs. Red color represents higher connectivity values for pre-condition compared to post-condition and blue color illustrates lower connectivity values for pre-condition compared to post-condition. ROIs included: lMFG, Left Middle Frontal Gyrus; rMFG, Right Middle Frontal Gyrus; lIFG, Left Inferior Frontal Parstriangularis; rIFG, Right Inferior Frontal Parstriangularis; rmOrbG, Right Medial Orbito Frontal Gyrus; lACCr, Left Rostral Anterior Cingulate; rACCr, Right Rostral Anterior Cingulate; lOrbG, Left Lateral Orbito Frontal Gyrus; rIFGorb, Right Inferior Frontal Orbital; lIFGop, Left Inferior Frontal Gyrus Opercular; lSTG, Left Superior Temporal Gyrus; rPreCG, Right Precentral Gyrus; rSTG, Right Superior Temporal Gyrus; lMTG, Left Middle Temporal Gyrus; rPoCG, Right Poscentral Gyrus; lPHG, Left Parahipocampal Gyrus; rSMG, Right Supramarginal Gyrus; lSMG, Left Supramarginal Gyrus; lPCUN, Left Precuneus; lstroke patientsL, Left Superior Parietal Lobule; rstroke patientsL, Right Superior Parietal Lobule; rLING, Right Lingual Cortex; rCAL, Right Calcarine; lMOG, Left Lateral Occipital Gyrus; rMOG, Right Lateral Occipital Gyrus.

When assessing the possible FC differences between stroke patients' conditions, a clear pattern of widespread increased FC within the beta band was found. Stroke patients showed significantly (*p* < 0.005) higher FC in the post-condition compared to the pre-condition in a variety of links comprising intra and inter-hemispheric, and antero-posterior long-range connections (Figure 4).

Moreover, when assessing the possible differences in FC between groups (post-condition and healthy controls) we did not find any statistically significant differences. This result indicated that the original FC disruption in the beta band was restored in stroke patients who went through the NR.

No significant results were found in other frequency bands in the pre and post comparison after FDR correction (*p* < 0.005). Nevertheless, there is a clear pattern of enhanced connectivity in low frequency bands (delta and theta) in the pre stage when compared with the brain activity of stroke patients recorded after the rehabilitation when a less restrictive statistical threshold was used (*p* < 0.05). Detailed description of these results could be found in the Supplementary Material, Appendix 3. These results were not included in the main findings of the present study because we wanted to focus on the most reliable FC signature, keeping the *p* < 0.005 value as the go/no go statistical limit.

### Correlations Between the Brain and Cognitive Recovery Patterns

With the aim to explore if FC changes were related with the enhanced cognitive performance in the stroke patients' group, we firstly calculated a ratio considering the strength of each functional link that differed between both conditions (FC ratio = Post/Pre). Then, we averaged these FC link ratios in just one value for each stroke patient. This provided a unique FC marker for each patient that condensed the information obtained by the whole network and the two MEG sessions. Next, for cognitive scores, we calculated the performance differences (D) for the most representative tests of each neuropsychological domain between pre and post conditions in the stroke patients' group (D = Post–Pre), with the aim of finding the strongest cognitive improvement, to reduce the redundancy of the information (since several scores measured the similar aspects of the same cognitive domain) and to avoid the statistical pitfall of multiple comparisons. Regarding the selection of the most representative scores included for the correlation analyses, the neuropsychological experts' team choose: *Functional Performance* (DEX-F), *Executive functions* (WCST-Persevering, Tower of Hanoi-3D-T), *Attention* (BTA), *Episodic memory* (WMS-III-LM1), *Working memory* (Digit span test), and *Language* (BNT). Moreover, the WAIS-III general indexes and some WAIS-III cognitive indexes were included (FIQ, PIQ, VIQ, PSI, POI). We finally included for the Spearman's correlation analyses the average FC strength ratio and 12 neuropsychological scores differences, illustrative of the cognitive improvement, for each stroke patient.

In order to facilitate the understanding of each patient cognitive improvement an extended material about individual neuropsychological performance was added in Appendix 2 in Supplementary Material. There, the punctuations for each patient before and after the rehabilitation are described in detail for those tests included in the present correlation analysis.

**Figure 5:**
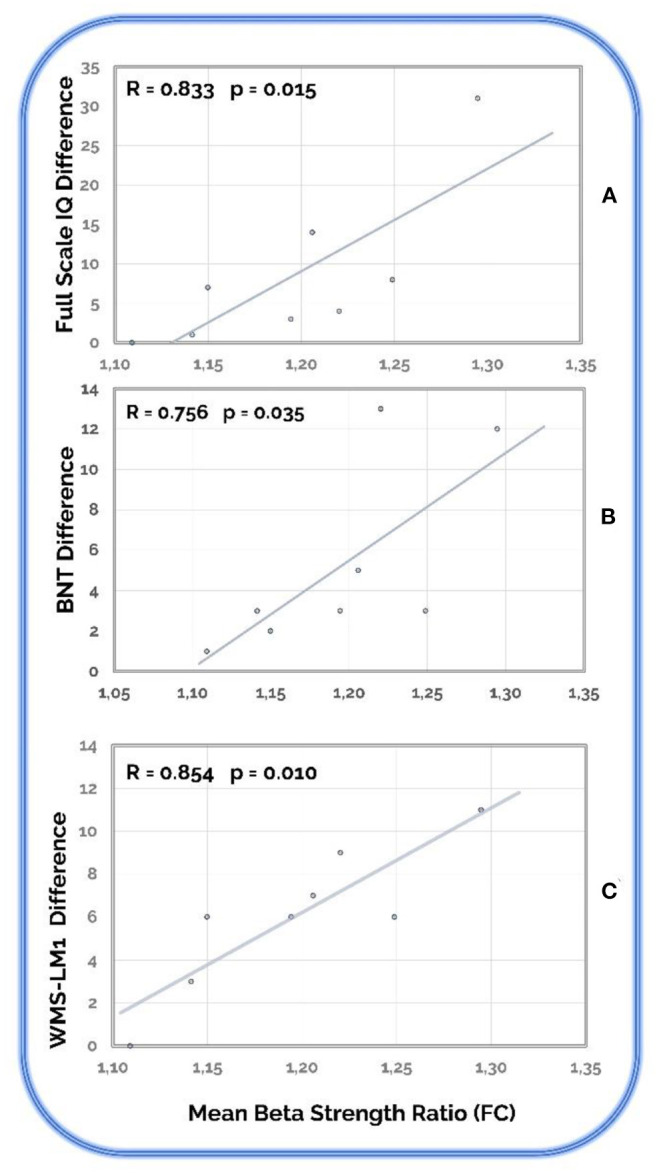
Recovery signatures correlations. Statistically significant correlations between cognitive differences and the mean beta strength ratio in the stroke patients' group. **(A)** Full Scale IQ (of WAIS-III); **(B)** BNT (Boston Naming Test); **(C)** WMS-LM1 (Logical Memory 1 of WMS-III).

We found three positive and significant recovery signatures correlations between the FC strength ratio and three cognitive measures: Full Scale IQ of WAIS-III (*R* = 0.833; *p* = 0.015), BNT (R = 0.756_;_ *p* = 0.035), and LM1 of WMS-III (*R* = 0.854; *p* = 0.010) (Figure 5).

### Prediction of Brain FC Recovery Based on Cognitive Performance After Stroke

Lastly, with the aim of exploring the predictive capacity of the neuropsychological test scores and the brain network recovery, we correlated the cognitive scores of the pre-condition and the FC strength ratio (by using Spearman correlation analyses).

**Figure 6:**
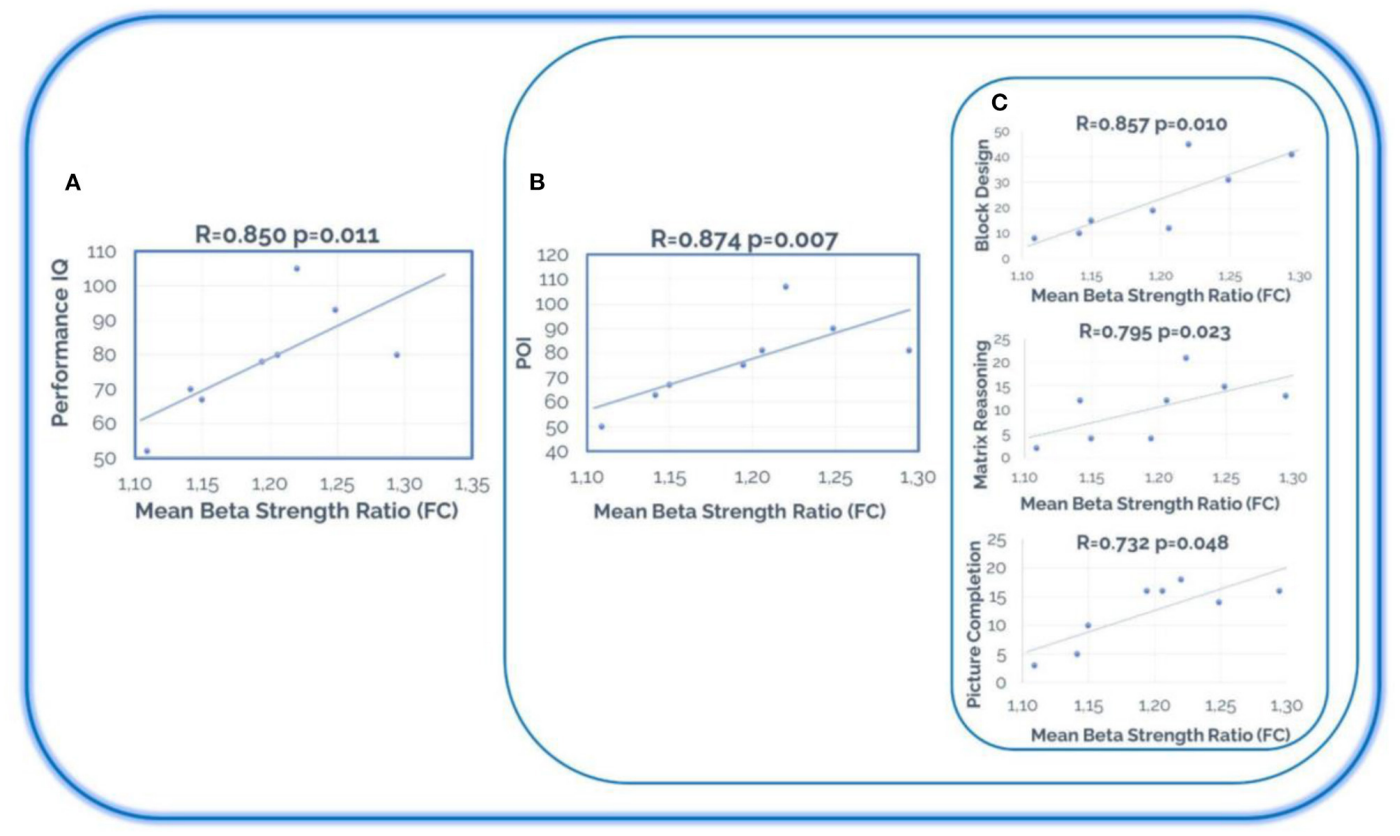
Recovery prediction. Statistically significant differences between the cognitive scores in the pre-condition and the mean beta strength ratio (post/pre) in the stroke patients' group. **(A)** Performance IQ (WAIS-III); **(B)** POI (Perceptual Organization Index of WAIS-III); **(C)** Block Design, Matrix Reasoning, and Picture Completion (of WAIS-III). Note that the tests represented in this figure are related to each other, since Block Design, Matrix Reasoning, and Picture Completion are the tests included in Perceptual Organization Index, and POI (Perceptual Organization Index) is included in Performance IQ (on the WAIS-III scale).

Thus, we observed two markers for recovery prediction in two global cognitive domains: (1) PIQ, with a significant positive correlation between FC strength ratio and the PIQ scores (*R* = 0.850, *p* = 0.011); (2) Perceptual Organization, with a significant positive correlation between FC strength ratio and the POI scores (*R* = 0.874, *p* = 0.007). Furthermore, within POI, we found a positive association with Picture Completion (*R* = 0.732; *p* = 0.048), Matrix Reasoning (*R* = 0.795; *p* = 0.023), and Block Design (*R* = 0.857; *p* = 0.010) (Figure 6).

## Discussion

The present study aimed to provide evidence of the neurophysiological mechanisms underlying cognitive deficits and changes in brain function associated with the recovery of cognitive processes in stroke patients who underwent a NR. Additionally, this study is focused on the exploration of the nature of the relationships between neurophysiological and neuropsychological changes.

In this sense, our results indicate a positive effect in acute stroke patients who received cognitive rehabilitation on both levels, the cognitive system and brain functioning. Nevertheless, the lack of a clinical control group (i.e., stroke patients without rehabilitation) did not allow us to make causality assumptions, assuring that cognitive improvement is due specifically or uniquely to cognitive rehabilitation because it could also represent some degree of spontaneous clinical recovery after stroke. Then, according to the results of the present study, stroke patients who undertook rehabilitation significantly improved their performance in 72% of cognitive and functional scores. But we also found than in neuropsychological scores related to specific cognitive domains such as executive functions, attention, language, episodic, and working memory, stroke patients showed a relative improvement (46% improved but there were significant differences between the post-condition and the control group), or even a total enhancement (26% improved, and there were no differences between post-condition and control group). These two degrees of positive changes were also found in scores related to global cognitive functioning such as Full-Scale IQ, PIQ, Speed Processing Index, Working Memory Index, or POI. In addition, some indicators related to functional performance, such as DEX or PCRS (completed by relatives of patients), also improved after rehabilitation. This trend could represent a partial cognitive and functional improvement and may have clinically relevant implications, since it may be considered as an indicator of recovery. To rule out the possibility that the learning effect could be influencing the improvement of some cognitive scores, we have other complementary data related to changes in brain function. Specifically, the stroke patients of this study exhibited a widespread increased FC pattern within the beta band, indicating that their original disruption was restored in the recording performed after NR in that frequency band. We also obtained two very important results associated with the relationships between cognitive scores and changes in FC. On the one hand, we found three positive and significant recovery signatures correlations between the FC strength ratio and three cognitive measures changes (in Full-Scale IQ, BNT, and LM1), and on the other hand, we observed the predictive capacity of some neuropsychological test scores (in the pre-condition) and the recovery of the brain network (in the FC strength ratio). In this sense, we found two predictive markers of brain recovery related to two global cognitive domains, PIQ and Perceptual Organization (both from the WAIS-III scale).

Based on these data, we can affirm that these stroke patients experienced at least some recovery in their global cognitive capacity, despite the different etiology and location of their lesions. Nonetheless the previous literature about the effect of NR on specific cognitive domains remains unclear. Low to moderate effects of rehabilitation in executive functions (6), attention (4, 52), or memory (53) have been reported. These results could have low consistency for different reasons: (1) the low methodological quality or insufficient description (2, 4, 52) the use of small samples; (3) the absence of comparisons between intervention and no intervention or placebo conditions (4, 6) the deficit of randomized control trials (4, 5, 52) the need for standardized definition and outcome measures (53, 54); and (6) the lack of inclusion of functional ability measures in the rehabilitation outcome evaluation (52). It seems important to find the most effective procedures to try recovering the cognitive deficits associated with stroke, considering that the prevalence of post-stroke cognitive impairment is 53.4% (55), since it causes an increase in the institutionalization rate and costs of care (56, 57) and a decrease in the quality of life (58). In addition, if stroke is a central factor in the development of cognitive impairment, or if this depends on the severity, subtype, location, or its recurrence, it becomes essential to understand the brain mechanisms that produce both deficits and their recovery. There is agreement around the idea that cognitive rehabilitation interventions aim to improve the impaired brain functions in stroke patients, and that it must be related to the damaged anatomical substrate (10). Usually, rehabilitation facilitates the development of behavioral and cognitive strategies that have a positive impact on the structural and functional recovery of the brain (53, 59). In this sense, it seems worthy to have in mind other promising complementary interventions including for example non-invasive transcranial magnetic stimulation to enhance some cognitive recoveries in stroke patients (60).

Restoration interventions aim to regain the cognitive abilities of stroke patients, including domain-specific interventions and treatments for generalized cognitive impairment (10). The patients of our study received an integrated treatment based on the holistic-comprehensive model proposed by Ben-Yishay and Diller (41), which is consistent with the interventions suggested by some experts in post-stroke cognitive rehabilitation in terms of their global treatment approach. This type of treatment could be very successful for this type of patients, insofar as it produces more clearly a pattern of overall improvement, both at behavioral and brain level. Other types of cognitive interventions, such as computer-assisted cognitive rehabilitation that has increased in recent years, although show some efficacy in improving attention, memory, executive function, or visuo-spatial neglect in stroke patients (61, 62), present very limited effects on working memory and even no effects on cognitive function compared to healthy controls (63).

As discussed above, overall review studies on the effectiveness of cognitive intervention with stroke patients do not provide clear conclusions. However, we must know that one of the most important issues regarding the functioning of the human mind has to do with the factor of interdependence between the different cognitive domains. This aspect is often overlooked in cognitive performance studies, and review studies of the effectiveness of cognitive treatments do not usually consider it. For example, there are studies that focus on improving attention after having specifically trained it, and thus with the rest of cognitive domains, without evaluating the impact of attention deficit or executive deficit in other domains such as memory or language. However, cognitive interdependence makes it very exceptional for patients who have brain injuries to suffer a specific cognitive deficit in a specific cognitive domain. The cognitive deficit of brain injury patients usually affects several domains, for example, visuo-spatial attention, working memory, executive functions, and episodic memory. Thus, trying to understand functioning of human cognition from independent cognitive domains, is probably an incorrect approach that hinders the interpretation of the results in neuropsychology. Usual intervention in the clinical setting is not as domain specific as studies suggest, since isolating cognitive processes in habitual actions is not easy. However, the neuropsychological literature continues to try to understand the effect of rehabilitation on each cognitive domain individually. This discrepancy requires a revision and a paradigm shift.

Furthermore, our intention was to go one step further trying to understand whether this recovery process seen at a cognitive and behavioral level could have some reflection in brain functioning. Cognitive functions depend on the integrated functioning of large-scale distributed brain networks (64). Specifically, recent evidence suggests that FC between brain regions may play an important role when difficulties arise from deficits in attention, memory, or other cognitive functions (65). In this context, we firstly looked for a FC pattern related to stroke. We found a disruption in the pattern of brain functioning, with a significant decrease in the beta band FC for intra and inter-hemispheric connections in stroke patients before rehabilitation compared to healthy controls. General disruption of dynamic networks after stroke have been previously reported in MEG studies (25). Alterations in beta band activity have been especially related to stroke compared to healthy controls (26), supporting our results.

On the other hand, understanding the interaction between brain regions within a network (i.e., their FC), and the interactions among networks are both important for efficient cognitive function (66). Therefore, exploring the possible neurophysiological mechanisms underlying the recovery process after stroke seems to be a crucial point in understanding the effect of cognitive rehabilitation on the brain. By observing stroke patients before and after the rehabilitation, a specific brain recovery pattern emerged, characterized by a widespread increased FC in the beta in the post-condition. The functioning of the beta frequency band has previously been related, in healthy population, to different cognitive tasks such as working memory (67–69), attention (70), and motor performance (71). On the contrary, our neurophysiological data were acquired during resting state (RS) which has been shown that is the most stable condition across patients with different symptomatology and it also has been considered a hallmark for clinical diagnosis and monitoring the recovery of patients that underwent a rehabilitation, both in MRI (18, 72) and MEG studies (22, 73). Previous results have described the role of RS FC as a predictor of motor learning ability in beta-band for healthy participants (74) or as a predictor of post-stroke motor recovery in alpha-band (75). Furthermore, the reorganization in FC in the beta-band during resting-state has previously been associated with the success of cognitive and physical interventions (13, 76, 77).

Up to this point, two independent markers of functional recovery (i.e., cognitive, and neurophysiological) were found in our stroke patients who went through the NR, but we developed further analyses to discover the possible relation between them. As mentioned before, trying to understand complex systems such as human cognition or brain functioning focusing on only some of their components gives partial information of the entire process. Therefore, with the aim to simplify the dimensionality of the data and to explore relationships between cognitive and neurophysiological findings, the difference (D = Post–Pre) of the most representative cognitive scores and the ratio of change (post/pre) of the total FC beta network strength were used. Three positive correlations between recovery signatures (i.e., cognitive and neurophysiology) were observed for stroke patients, corresponding to Full Scale IQ of WAIS-III, Boston Naming Test, and Logical Memory 1 of WMS-III. These results showed that the beta connectivity changes after the NR, compared with the data obtained in the first recording are, in fact, the reflection of the cognitive improvement in the brain. The measures related with improvement represent global neuropsychological indices, which contain different cognitive domains such as sustained and switching attention, visuo-spatial attention, visuo-spatial working memory, planning, flexibility, or processing speed, but also episodic memory or verbal denomination. Initially, the neuropsychological deficit observed in stroke patients was global and their subsequent cognitive recovery, although not complete, was also general. The scope of cognitive changes after NR was really wide, probably due to the type of intervention, which was holistic and not only focused on specific cognitive functions, including global and interdependent domains, and focusing on individual cognitive, functional, emotional, and behavioral imbalances. All these evidences are consistent with the brain network global changes observed in stroke patients.

While addressing brain and cognitive changes in stroke patients is important to understand the underlying mechanisms of stroke and brain plasticity, the early detection of patterns or biomarkers is also relevant to predict which subjects are more likely to improve and benefit from neurorehabilitation. In this regard, adjustments can be made for those patients who will not benefit from this option. Thus, we performed an additional correlation analysis in which the pre-condition cognitive measures and the ratio of change (post/pre) of the total FC beta network strength were taken into account. Two global cognitive markers were stated as predictors of brain functioning recovery, PIQ and POI. Furthermore, within POI, we found a significant association for every subtest included in the global index (i.e., Picture Completion, Matrix Reasoning, and Block Design). In the clinical setting, the role of prediction in terms of the degree of future recovery has always been important, however it is a complex and complicated issue. Until now we only had clinical, cognitive, behavioral, and social variables, but these results indicate that the relationship between behavior and the brain can contribute to this topic. In this case, the results indicate that the initial state around some cognitive domains such as visuo-spatial attention, visuo-spatial working memory, or planning capacity, could have a very relevant role in the evolution and recovery of the brain network of stroke patients. This information is not only important to predict the patients who will improve the most, but it can also serve to think about more powerful intervention procedures for those patients who have a more serious deficit around these cognitive domains. This result has very relevant clinical implications.

In conclusion, if NR aims to improve people's cognitive function in order to restore their general performance and independence in functional activities, the results of the present study are in line with this objective, showing a clear improvement pattern in stroke patients who received NR both cognitively and brain function. We are also sure that this study points out the importance of including neuropsychological and neurophysiological variables in the assessment of the outcome and effectiveness of psychological interventions of stroke patients.

## Limitations and Future Research

An important contribution of this study may be that, unlike most studies, functional brain connectivity was measured with MEG. Despite the intrinsic limitations of BOLD fMRI, MEG is a measure of brain activity with incredibly high temporal resolution (ms). Despite its advantages, MEG is an underused neuroimaging tool in clinical and research contexts. Although the sample of this exploratory study size was small, we were able to identify a pattern of recovery of FC in the beta band related to cognitive enhancement in stroke patients who underwent a NR. Additionally, we were able to make predictions based on the cognitive performance of stroke patients before rehabilitation about the future functional restoration of the brain network. Thus, larger MEG studies with stroke patients are needed to demonstrate the power of this neurophysiological tool within this neurological field. Another limitation that this study faces is the heterogeneity of stroke patients, in terms of etiology and location of the injury. However, we believe that this heterogeneity has provided an interesting approach to the study since it has allowed us to explore the effect of cognitive intervention both at cognitive and neurophysiological level in stroke patients with different etiology. Furthermore, another limitation of the study is the absence of a clinical control group (i.e., stroke patients without rehabilitation). Considering this limitation, we cannot assure that cognitive improvement is due specifically or uniquely to cognitive rehabilitation because it could also represent some degree of spontaneous clinical recovery after stroke. In accordance with the aforementioned obstacles, future studies should include a group of stroke patients without cognitive intervention (e.g., on the waiting list), and larger samples of patients that allow comparisons based on the etiology and the location of the lesion. Finally, it would be interesting to focus on different networks of the brain (78) such as the default mode, the salience, or the executive control networks (79), to explore specific changes in each network recovery pattern, and its possible relationship to particular improvements in cognition after stroke.

## Data Availability Statement

The raw data supporting the conclusions of this article will be made available by the authors, without undue reservation.

## Ethics Statement

The studies involving human participants were reviewed and approved by the National Brain Injury Rehabilitation Center Ethics Committee (Madrid). The patients/participants provided their written informed consent to participate in this study.

## Author Contributions

SP, LT-S, ML, NP, and FM designed research. SP and LT-S performed main calculations of the study and prepared figures. SP, LT-S, ML, BC, LC, AB, FM, and NP collaborated actively in writing the manuscript. All authors contributed to the article and approved the submitted version.

## Funding

Financial support of the project was provided by IMSERSO (07-2008) and the Spanish MICINN (PSI2011-28388). Research by SP was supported by the Spanish MINECO post-doctoral fellowship (FJC2019-041205-I). Additionally, this work was supported by a predoctoral researcher grant from Universidad Complutense de Madrid (CT42/18-CT43/18) and co-founded by Santander Bank to LT-S, and by the National Council of Science, Technology and Technological Innovation (CONCYTEC, Perú) through the National Fund for Scientific and Technological Development (FONDECYT, Perú) to BC.

## Conflict of Interest

The authors declare that the research was conducted in the absence of any commercial or financial relationships that could be construed as a potential conflict of interest.

## Publisher's Note

All claims expressed in this article are solely those of the authors and do not necessarily represent those of their affiliated organizations, or those of the publisher, the editors and the reviewers. Any product that may be evaluated in this article, or claim that may be made by its manufacturer, is not guaranteed or endorsed by the publisher.
