# Supplementary material for: Resting-State Beta-Band Recovery Network Related to Cognitive Improvement After Stroke
Source: Front Neurol. 2022 Feb 25;13:838170. doi: 10.3389/fneur.2022.838170 (PMC8914082; doi:10.3389/fneur.2022.838170)
Supplement: Supplementary file 1 [file Data_Sheet_1.docx]

**SUPPLEMENTARY MATERIAL**

**Title:** Resting-state beta-band recovery network related to cognitive improvement after stroke

Appendix 1

Table S1. Abbreviations of the 68 regions of interest based on the Brainstorm atlas Desikan-Killiany, and their corresponding translation to the Talaira.

| **Abbreviation** | **Desikan-Killiany Regions** | **Translation to Talairach and Broadmann areas** |
| --- | --- | --- |
| ACCc | Caudal Anterior Cingulate | Limbic Lobe, Cingulate Gyrus,24 |
| ACCr | Rostral Anterior Cingulate | Limbic Lobe, Anterior Cingulate, 32 |
| CAL | Pericalcarine | Occipital Lobe, Cuneus, 17 |
| CUN | Cuneus | Occipital Lobe, Cuneus, 18 |
| EC | Entorhinal Cortex | Limbic Lobe, Entorhinal Cortex, 28 |
| FFG | Fusiform Gyrus | Fusiform Gyrus, 20 |
| HES | Transversetemporal | Temporal Lobe, Superior Temporal Gyrus,41&42 |
| IFGop | Parsopercularis | Frontal Lobe, Inferior Frontal Gyrus, 44 |
| IFGorb | Parsorbitalis | Frontal Lobe, Inferior Frontal Gyrus, 47 |
| IFGtri | Parstriangularis | Frontal Lobe, Inferior Frontal Gyrus, 45 |
| INS | Insula | Sub-lobar, Insula, 13 |
| IPL | Inferior Parietal Lobule | Inferior Parietal Lobule, 39 |
| ITG | Inferior Temporal Gyrus | Temporal Lobe, Inferior Temporal Gyrus, 20 |
| LING | Lingual Gyrus | Occipital Lobe, Lingual Gyrus, 18 |
| MCC | Posterior Cingulate Gyrus | Limbic Lobe, Cingulate Gyrus, 24 |
| MFGc | Caudal Middle Frontal Gyrus | Frontal Lobe, Middle Frontal Gyrus, 6 |
| MFGr | Rostral Middle Frontal Gyrus | Frontal Lobe, Middle Frontal Gyrus, 10 |
| MOG | Lateral Occipital Gyrus | Occipital Lobe, Middle Occipital Gyrus, 18 |
| MTG | Middle Temporal Gyrus | Temporal Lobe, Middle Temporal Gyrus, 21 |
| MTGb | Bankssts | Temporal Lobe, Middle Temporal Gyrus, 22 |
| OrbG | Lateral Orbito Frontal Gyrus | Frontal Lobe, Orbital Gyrus, 11 |
| OrbG | Medial Orbito Frontal Gyrus | Frontal Lobe, Orbital Gyrus, 11 |
| PCC | IsthmusCingulate Gyrus | Limbic Lobe, Posterior Cingulate, 31 |
| PCL | Paracentral Lobule | Frontal Lobe, Paracentral Lobule, 6 |
| PCUN | Precuneus | Parietal Lobe, Precuneus, 7 |
| PHG | Parahippocampal | Limbic Lobe, Parahippocampal Gyrus, 36 |
| PoCG | Postcentral Gyrus | Parietal Lobe, Postcentral Gyrus, 1 |
| PreCG | Precentral Gyrus | Frontal Lobe, Precentral Gyrus, 4 |
| SFG | Frontal Pole | Frontal Lobe, Superior Frontal Gyrus, 10 |
| SFG | Superior Frontal Gyrus | Frontal Lobe, Superior Frontal Gyrus, 8 |
| SMG | Supramarginal Gyrus | Parietal Lobe, Supramarginal Gyrus, 40 |
| SPL | Superior Parietal Lobule | Parietal Lobe, Superior Parietal Lobule, 7 |
| STG | Superior Temporal Gyrus | Temporal Lobe, Superior Temporal Gyrus,22 |
| TPO | Temporal Pole | Temporal Lobe, Superior Temporal Gyrus, 38 |

Appendix 2

Table S2. Neuropsychological scores for stroke patients before and after the cognitive rehabilitation. For each test and patient, pre and post neuropsychological scores separated by a slash are shown. The 12 tests presented here were chosen by the neuropsychological experts’ team.

|  | **Functional Performance** | **Executive Function** | | **Attention** | **Language** | **Episodic Memory** | **Working Memory** | **WAIS-III indexes** | | | | |
| --- | --- | --- | --- | --- | --- | --- | --- | --- | --- | --- | --- | --- |
| **Patient** | DEX-F | WCST- Pers. | Hanoi Tower (3-T) | BTA | BNT | Logical Memory I  (WMS) | Digit span  (WAIS-III) | FIQ | VIQ | PIQ | PSI | POI |
| 1 | 42/12 | 27/33 | 81/27 | 8/19 | 51/54 | 33/39 | 15/14 | 87/90 | 97/98 | 78/83 | 89/92 | 75/77 |
| 2 | 65/39 | 55/33 | 119/48 | 2/3 | 53/54 | 15/15 | 14/13 | 71/71 | 97/98 | 52/50 | 57/57 | 50/50 |
| 3 | 6/9 | 6/5 | 119/39 | 8/10 | 59/60 | 29/36 | 13/14 | 101/108 | 105/110 | 97/105 | 70/75 | 105/117 |
| 4 | 16/2 | 8/11 | 373/29 | 7/11 | 56/58 | 22/28 | 11/17 | 84/91 | 102/107 | 67/73 | 75/84 | 67/75 |
| 5 | 33/12 | 55/45 | 95/16 | -/- | 0/27 | 30/25 | -/- | -/- | -/- | 97/97 | 84/87 | 105/105 |
| 6 | 18/18 | 25/14 | 202/48 | 7/16 | 37/49 | 6/17 | 5/16 | 69/100 | 62/97 | 80/105 | 65/84 | 81/101 |
| 7 | 5/5 | 27/6 | 98/45 | 14/18 | 49/52 | 18/24 | 18/21 | 100/108 | 106/110 | 93/106 | 109/117 | 90/107 |
| 8 | 17/15 | 10/8 | 17/10 | 17/16 | 41/54 | 22/31 | 4/10 | 96/100 | 89/97 | 105/105 | 81/89 | 107/109 |
| 9 | 36/22 | 42/12 | 36/46 | 18/19 | 54/57 | 25/28 | 17/17 | 103/104 | 122/122 | 70/83 | 89/95 | 63/87 |
| 10 | 40/23 | 38/35 | 66/18 | 14/15 | 39/44 | 10/17 | 10/13 | 75/89 | 73/88 | 80/93 | 84/89 | 81/90 |

Appendix 3

The main finding of this study related to brain functioning changes after stroke was a diminished beta FC when compared patients in the pre stage with controls. When assess pre-post differences, in the patient’s group, we found the main network of this study, where beta FC seemed to be recovered. Result in other frequency bands did not survive the statistics (p < 0.005, FDR). Nevertheless, with a lower statistical threshold (p < 0.05, FDR) results in delta and theta frequency bands were observed.

As it can be seen in figure S1, there exists a clear pattern of enhanced connectivity in low frequency bands (delta and theta) in the pre stage when compared with the brain activity of stroke patients recorded after the rehabilitation. These results were not included in the main findings of the present study because we wanted to focus on the most reliable FC signature, keeping the p < 0.005 value as the go/no go statistical limit. Although, the delta/theta slowing is a brain marker well described in stroke literature (Kaltiainen *et al.*, 2018; Ip *et al.*, 2019; Cassidy *et al.*, 2020), we considered that the novelty and utility of these results for the current knowledge were lower. Notwithstanding, the existence of these results augmented the trust on the main findings in beta band, giving strength to fact that our stroke sample, in fact, follows the common patter of slowness.


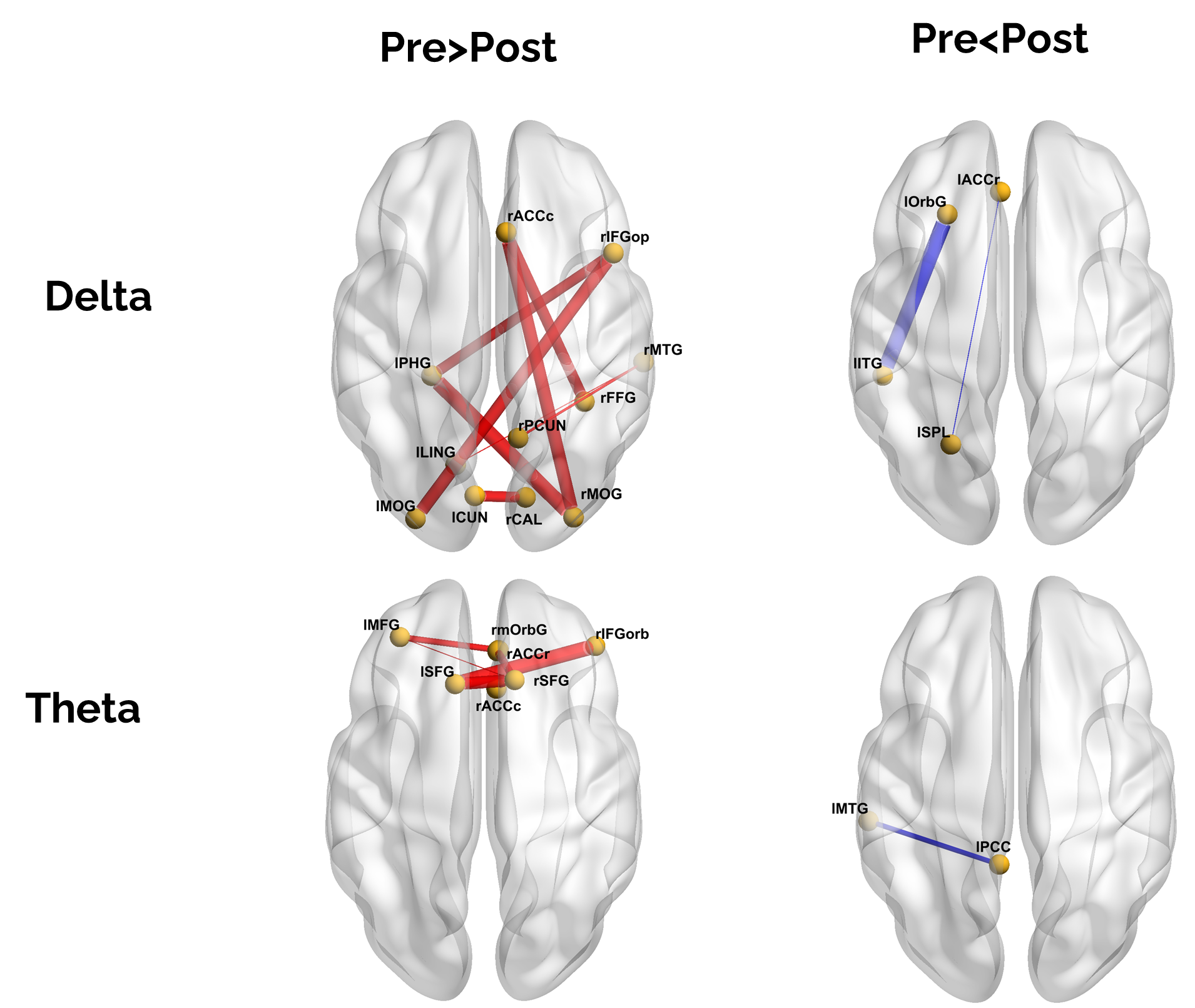


Figure S1. Slowness pattern of the FC in stroke patients. . Top-panel) FC delta results (p < 0.05, corrected) when comparing within the stroke patients’ group, the pre-condition and the post- condition. Line thickness of significant links is proportional to FC values (a higher value corresponds to thicker lines, and vice versa. Red color represents higher connectivity values for pre-condition compared to post-condition and blue color illustrates lower connectivity values for pre-condition compared to post-condition.

*ROIs included: lMFG: Left Middle Frontal Gyrus, lSFG: Left Superior Frontal Gyrus, rSFG: Right Superior Frontal Gyrus, rmOrbG: Right Medial Orbito Frontal Gyrus, rACCc: Right Caudate Anterior Cingulate, rACCr: Right Rostral Anterior Cingulate, lACCr: Left Rostral Anterior Cingulate, lPCC: Left Posterior Cingulate, lOrbG: Left Lateral Orbitofrontal Gyrus, rIFGorb: Right Inferior Frontal Orbital, rIFGop: Right Inferior Frontal Gyrus Opercular, lPHG: Left Parahipocampal Gyrus, rMTG: Right Middle Temporal Gyrus, lMTG: Left Middle Temporal Gyrus, rFFG: Right Fusiform Gyrus, rPCUN: Right Precuneus, lLING: Left Lingual, lMOG: Left Lateral Occipital Gyrus, rMOG: Right Lateral Occipital Gyrus, lCUN: Left Cuneus, rCAL: Right Calcarine, lITG: Left Inferior Temporal Gyrus, ISPL: Left Superior Parietal Lobule.*
